# 2D:4D Ratio and Autism Spectrum Disorder in Brunei Darussalam

**DOI:** 10.1007/s10803-021-04899-9

**Published:** 2021-02-11

**Authors:** Shirley H. F. Lee, Syahiirah Abd Aziz, Mawarni Hamid, Ya Chee Lim, David Koh, Li Ling Chaw

**Affiliations:** 1 PAPRSB Institute of Health Sciences, Universiti Brunei Darussalam, Jalan Tungku Link, Gadong, BE1410 Brunei Darussalam; 2 Present Address: School of Medicine, University College, College Road, Cork, T12 K8AF Ireland; 3 Ministry of Health, Commonwealth Drive, Bandar Seri Begawan, BB3910 Brunei Darussalam

**Keywords:** Autism spectrum disorders (ASD), 2D:4D ratio, Digit ratio, Extreme male brain (EMB), Hormones, Androgens, Testosterone, Brunei Darussalam, 2D:4D ratio symmetry

## Abstract

**Background:**

Despite the global increase in the prevalence of autism spectrum disorders (ASD), relevant research studies are lacking in Brunei Darussalam. Various studies have shown a significant association between a lowered 2D:4D ratio (ratio of second digit/index finger to the fourth digit/ring finger) and ASD, making it one of the potential phenotypic biomarkers for early detection of autism, which is important for early intervention and management.

**Objective:**

The objective of this study is to explore the association between 2D:4D ratio and ASD in Brunei Darussalam, as a potential tool to complement early ASD diagnosis.

**Methods:**

We conducted a case–control study comprising 28 ASD and 62 typically developing (TD) children in the case and control group, respectively (age range: 3–11 years old; median age: 6 years old). Median 2D:4D ratios were measured, compared and analysed between the two groups. Logistic regression models were used to explore potential associations between the median 2D:4D ratio and ASD in respective gender, for both left and right hands, independently.

**Results:**

Our study shows that the median 2D:4D ratio of left hand in ASD males is significantly lower than those in TD males, after adjusting for ethnicity and age [Odds Ratio (OR) = 0.57 (95% Confidence Interval (CI): 0.31–0.96); *p* = 0.044]. For females, there is no association of ASD with the median left hand 2D:4D ratio [OR = 3.09 (95% CI: 0.98–19.86); *p* = 0.144] or the median right hand 2D:4D ratio [OR = 1.23 (95% CI: 0.42–3.88); *p* = 0.702]. Our study also shows a significant positive correlation and/or a reduced asymmetry between the average 2D:4D ratio of left hands and right hands in ASD males (Pearson’s correlation (*r*) = 0.48; 95% CI: 0.076–0.75, *p* = 0.023).

**Conclusions:**

There is significant association between a lowered median 2D:4D ratio of the left hand (in males only) and ASD diagnosis. Once validated in a larger sample size, a lowered median 2D:4D ratio on the left hand may be a potential tool to complement ASD diagnosis for males in our study population. There is no association between the median 2D:4D ratio (left or right hands) and ASD in females, which could be due to the small female sample size and/or the possibility of different aetiology for ASD in females. Reduced asymmetry between the average 2D:4D ratio of left and right hands observed in ASD males only (not in ASD females) also suggests the importance of considering gender-specific biomarkers for ASD diagnosis.

**Supplementary Information:**

The online version contains supplementary material available at 10.1007/s10803-021-04899-9.

## Introduction

Autism spectrum disorders (ASD) or autism is a manifestation of developmental abnormalities of the brain (Landrigan 2010; Manning-Courtney et al. 2003). Globally, approximately 1 in 160 children has ASD; equivalent to a median prevalence of 62/10,000 (Elsabbagh et al. 2012; WHO 2019). In most cases, classical traits of individuals with ASD include delayed speech development, lack of social interaction as well as repetitive behaviours and restricted interest (Levy et al. 2009). ASD or “autism” in this context refers to those diagnosed according to Diagnostic and Statistical Manual of Mental Disorders, 5th Edition: DSM-5 (DSM-V), and/or Autism Diagnostic Observation Schedule (ADOS). For consistency, ASD will be used thereafter.

Diagnosing ASD in children can be quite challenging due to heterogeneous clinical features expressed by individuals. However, the role of healthcare professionals in assessing the development and behaviour of children, along with the history provided by the parents or the primary caregiver (as well as teachers) in reporting the child’s usual behaviour and ability, are crucial in diagnosis. Common diagnostic tools involve checklists or questionnaires including Autism Diagnostic Observational Schedule (ADOS), and the screening tool for ASD in toddlers and young children (STAT) among others (Huerta and Lord 2012; Vllasaliu et al. 2016). Currently, there is no cure for ASD. Majority of research focus on early diagnosis and management of ASD symptoms such as psychosocial therapy and medication to improve the quality of life of individuals with ASD (DeFilippis and Wagner 2016; Park et al. 2016; Mackus et al. 2017; Auyeung et al. 2010). The aetiology of ASD is complex, where multiple factors are involved. Abnormalities in certain brain regions such as the amygdala, prefrontal cortex and nucleus accumbens are among denoted factors (Park et al. 2016). Studies have also implicated genetic factors based on concordance rate from twin studies (Bailey et al. 2009) and environmental factors such as maternal intrauterine environment during the prenatal period (London 2000). In addition, advanced parental age (maternal and paternal), abnormal gestational age (pre-term or post-term infant), low birth weight of the infant and the mode of delivery of the infant by caesarean section (CS) were among suggested risk factors for ASD (Curran et al. 2015; Karimi et al. 2017; Parner et al. 2012). Both genetic and environmental factors may interact to influence the manifestation of the disorder (Tordjman et al. 2014).

Various studies have reported a gender bias in ASD, with a male predominance. Globally, the ratio of male-to-female affected with ASD ranges from 3:1 to 16:1 (Loomes et al. 2017; Fombonne 2009). In Brunei, this ratio stands at approximately 3.5:1 (personal communication; unpublished data). Although recent studies have suggested that some of the current diagnostic criteria used to diagnose ASD is gender-biased (Haney 2016; Beggiato et al. 2017), leading to under-diagnosis in females, several theories have been put forward to explain the male gender bias in ASD, such as the Extreme Male Brain (EMB) theory (Baron-Cohen 2002). This theory suggests that excess foetal steroid hormone exposure in particular, excess foetal testosterone (FT) in the maternal intrauterine environment may influence structural as well as functional development of the brain in utero, leading to abnormal maturation of the brain, similar to those observed in autistic individuals. Consequently, hyper-masculinised traits are expressed in ASD individuals.

Interestingly, high levels of testosterone in males were found to be associated with a lower 2D:4D ratio. The 2D:4D ratio refers to the ratio of the second digit/index finger to the fourth digit/ring finger in an individual. It is considered a sexually dimorphic trait for both the left and right hands, where the ratio tends to be lower in males (mean = 0.98) than in females (mean = 1.00). This is due to the urinogenital system and fingers sharing a developmental link as both are regulated by *Hox* genes. This is presented from the age of 2 years and the difference is more prominent on the right hand (Manning et al. 1998). The length of the digits is determined on the 14th week of intrauterine foetal development. 2D:4D ratio is lowered when the prevailing androgen in utero is higher compared to the concentration of oestradiol such as oestrogen during the foetal period (Mackus et al. 2017; Manning et al. 1998). Therefore, the degree of foetal testosterone exposure in the maternal intrauterine environment is indirectly measurable by obtaining means of the 2D:4D ratio (Manning et al. 1998).

Several studies have reported positive associations between 2D:4D ratio and ASD. A case–control study conducted in the United Kingdom has also proven a significantly lowered 2D:4D ratio in children with ASD compared to TD children (Manning et al. 2001). Several independent studies conducted in Saudi Arabia, Slovakia and Thailand also support this finding (Al-Zaid et al. 2015; Krajmer et al. 2011; Noipayak 2009). Furthermore, a stronger negative correlation of 2D:4D ratio and autistic traits among females with ASD has also been observed (Bruin et al. 2009).

A higher number of autistic individuals are born to mothers with Polycystic Ovarian Syndrome (PCOS) (Manning et al. 1998), an endocrine disorder which affects approximately 5% to 15% of females of reproductive age. Women with PCOS commonly exhibit higher than normal levels of testosterone and to some extent hirsutism (Manning et al. 2001; Al-Zaid et al. 2015). Hirsutism on the other hand, is a condition resulting from hyperandrogenism. A subsequent study has shown a positive association of maternal hirsutism with ASD offspring (Krajmer et al. 2011).

There is great concern about the increasing prevalence of ASD in Brunei. Data from Development Paediatric Services (DPS), Ministry of Health have shown that from the year 1986 to 2017, 864 individuals (674 males and 190 females) have been diagnosed with ASD in Brunei Darussalam (personal communication; unpublished data). As of January to September 2019, about 176 new ASD cases have been diagnosed, equivalent to about 5 cases per week (personal communication; unpublished data). Besides DPS, there are various private healthcare providers, ASD-support groups and non-governmental organisations (NGOs) in the country (LaVida, Learning Ladders, Little Bosses IQDemy and SMARTER, among others).

Despite the high number of ASD cases in Brunei, research studies are unfulfilled. Given the challenges in diagnosing ASD early and the accumulating evidence on the association between 2D:4D and ASD, we are interested to investigate this based on the local context. Thus, the objective of this study is to investigate the association between 2D:4D ratio and ASD in the Bruneian population. A significant association between the 2D:4D ratio and ASD in the Bruneian population may serve as a tool to complement diagnosis to facilitate early intervention and management of symptoms in individuals with ASD.

## Methods

### Study Design

We conducted a case–control study, where ASD and TD children between 3 and 12 years old were recruited as our cases and controls, respectively. The inclusion criteria for the ASD group include: (i) Children with Bruneian citizenship or permanent residency (PR) within the age range 3 to 12 years old (inclusive) diagnosed with ASD at specialist centres (local or abroad) and (ii) Children registered at DPS. For the TD group, the inclusion criterion is children with Bruneian citizenship or PR between 3 and 12 years old (inclusive) not diagnosed with ASD and/or any other form of learning disability (local or abroad). The inclusion criterion common to both ASD and TD groups is children with five uninjured fingers on both hands. Meanwhile, the exclusion criteria include: (i) Children with less than or more than 5 fingers and (ii) Children with injured finger(s) on any hand. Due to logistic and resource issues, recruitment was done only in Brunei-Muara district, the most populated district in Brunei Darussalam where 69.6% of the population resides (2017).

Community members including individuals with autism and their families, gatekeepers of autism NGOs (SMARTER and Little Bosses IQdemy), TD children from primary schools and the respective heads of schools and class teachers, as well as clinicians from Ministry of Health, Brunei Darussalam were involved in this research.

### Recruitment

Mothers of ASD and TD children were approached by teachers in the respective institutes and received information about the study through a participant information sheet. Mothers who agreed to enrol their children in the study completed written consent forms and online demographic questionnaires (which include questions about age, gender and ethnicity of their child) which were returned to respective teachers. Arrangements were made with the respective teachers for finger length measurements (through palm photos) to be obtained from the child/children of mothers who have consented to participate. All palm photos were obtained within the school compound by one researcher with a smartphone camera, with assistance from another researcher to provide instructions (placing hands ventral-side-up with both left and right hands laying against a flat surface with a plain white background) to students before palm photos were taken.

The ASD group consists of individuals diagnosed with ASD, who are registered at the DPS in Brunei Darussalam. They were recruited from non-governmental ASD-support organisations (SMARTER EDGE and Little Bosses IQDemy). In this study, all ASD diagnoses were performed locally in accordance to the Diagnostic and Statistical Manual of Mental Disorders, 5th Edition: DSM-5 (DSM-V), and/or Autism Diagnostic Observation Schedule (ADOS) by certified clinicians at DPS.

The TD group consisted of typically developing children who have never been diagnosed with ASD at any registered health centres (local or overseas), including DPS. They were recruited from three primary schools in the capital city (namely St Andrew’s, St George’s, and Sekolah Rendah Rimba I). These schools were selected to represent a range of students enrolled in both public and private schools. The non-response rate range from 45 to 53%.

### Data Collection

Mothers who agreed to enrol their children in the study were provided a questionnaire, to capture the demographic profile of their children (age, ethnicity, and gender of their child). The socioeconomic status and educational attainment levels of mothers and/or children were not reported/analysed due to a low response rate.

Photographs of the right and left hands were obtained and used for finger length measurements. As mentioned earlier, each participant was asked to place his/her hands ventral-side-up with both left and right hands laying against a flat surface with a plain white background (a blank white A4 sheet) to ensure precision in measurement. During palm-photo sessions, each participant was asked to straighten his/her fingers as much as possible. Consistency in the position of the smartphone’s camera was also maintained, where the photos were taken perpendicular to the plane or surface of the palms of each participant.

Digital photos were printed before finger length measurements were obtained. To ensure that the photo used for measurement is valid, the following criteria must be met: (i) straight fingers/digits from each participant (ii) clear fingertip borders from each participant, and (iii) clear and visible basal crease line for each finger/digit.

Each set of palm photo of a child is measured by two different raters, who were both double-blinded to the child’s condition, using a digital Vernier calliper (correct to 0.01 mm). In this study, finger/digit length is defined as the distance between the middle basal proximal crease of each finger and the mid finger-tip (Supplementary Fig. 1; S1). Written informed consent was provided the participant’s legally authorized representative (mother) to publish the palm photo in S1. From each palm photograph (S1), Raters 1 and 2 provided two and three 2D:4D readings respectively, giving a total of five 2D:4D readings for each hand. The 2D:4D ratio from each rater was calculated by dividing the average 2D value by the average 4D value for each hand. The final 2D:4D readings for each hand were obtained by averaging the values between the two raters.

### Statistical Analysis

The intraclass correlation coefficient (ICC) was calculated to assess the agreement between the 2D:4D ratios measured by two raters, for both left and right hands. The ICC value was very high for both hands (left hand = 0.98; right hand = 0.97), indicating high similarity between raters. Hence, the 2D:4D ratios from the two raters were averaged for each hand. As the correlation between the 2D:4D ratios between the left and right hands was low (r = 0.121, Pearson’s correlation), we decided to report the 2D:4D ratio separately for both hands.

Descriptive analyses using Mann–Whitney’s test and Fisher’s Exact tests were conducted to compare between the ASD and TD groups, as well as between both genders. Median 2D:4D ratios were reported because the variable was not normally distributed, particularly for the female population. Next, logistic regression analysis was done to determine any association between 2D:4D ratio and ASD diagnosis, after accounting for ethnicity and age. This is because both ethnicity (Manning et al. 2004) and age (Cho et al. 2015; Gillam et al. 2008) have been reported to influence the 2D:4D ratio. In addition, this analysis was done separately for each gender, considering the male preponderance nature in ASD and that 2D:4D ratio is sexually dimorphic (Guyatt et al. 2015).

To ensure clear comparison of the results for both genders, the 2D:4D ratio for each hand was standardised within each gender, using the following formula:

$$Standardised\,2D:4D\,ratio = \frac{Average\,of\,2D:4D\,ratio }{Standard\,deviation\,of\,2D:4D\, ratio}$$

This is done such that one unit change in the 2D:4D ratio represents a change by 1 standard deviation (SD). This impacts the interpretation of the logistic regression results, however, the raw 2D:4D ratios are reported in the descriptive table on the demographic characteristics of the study population (Table 1). Statistical analysis was performed using R (version 3.5.1). A p-value of < 0.05 was considered statistically significant.

**Table 1:** Demographic characteristics of the study population

Demographic characteristics	Total participants	ASD group (n = 28)	TD group (n = 62)	p-value
Median age in years (IQR)	6.0 (2.0)	6.0 (4.0)	6.0 (2.0)	0.798
Gender (n, %)				
Male	71 (78.9)	22 (31.0)	49 (69.0)	1
Female	19 (21.1)	6 (31.6)	13 (68.4)	
Ethnicity (n, %)				
Malay	68 (75.6)	20 (29.4)	48 (70.6)	0.728
Others	22 (24.4)	8 (36.3)	14 (63.6)	
Median 2D:4D ratio (IQR)				
Left hand	0.954 (0.084)	0.949 (0.078)	0.961 (0.085)	0.255
Right hand	0.947 (0.077)	0.951 (0.069)	0.943 (0.078)	0.61

*ASD* Autism spectrum disorder; *TD* Typically developing; *IQR* Interquartile range

## Results

We enrolled a total of 28 ASD and 62 TD children between January 2018 and March 2019. Their age range was between 3 and 11 years old (median = 6 years old, IQR = 2). About three quarters of them were male (78.9%, n = 71) and of Malay ethnicity (75.6%, n = 68). There was no significant difference in the demographic characteristics between the ASD and TD groups (Table 1). No significant difference was also observed when comparing the median 2D:4D ratios for each hand between the ASD and TD group (Table 1). However, the median 2D:4D ratio for the left hand was comparatively lower for the ASD group (median = 0.949, IQR = 0.078) when compared to that of the TD group (median = 0.961, IQR = 0.085) (Table 1).

There is no difference (*p* = 0.549) between the median 2D:4D ratios for the left hand of TD male children (0.962) and TD female children (0.938) (data not shown). Likewise, for the right hand, there is no difference (*p* = 0.303) between the median 2D:4D ratios for TD male children (0.940) and TD female children (0.996) (data not shown).

**Fig. 1:**
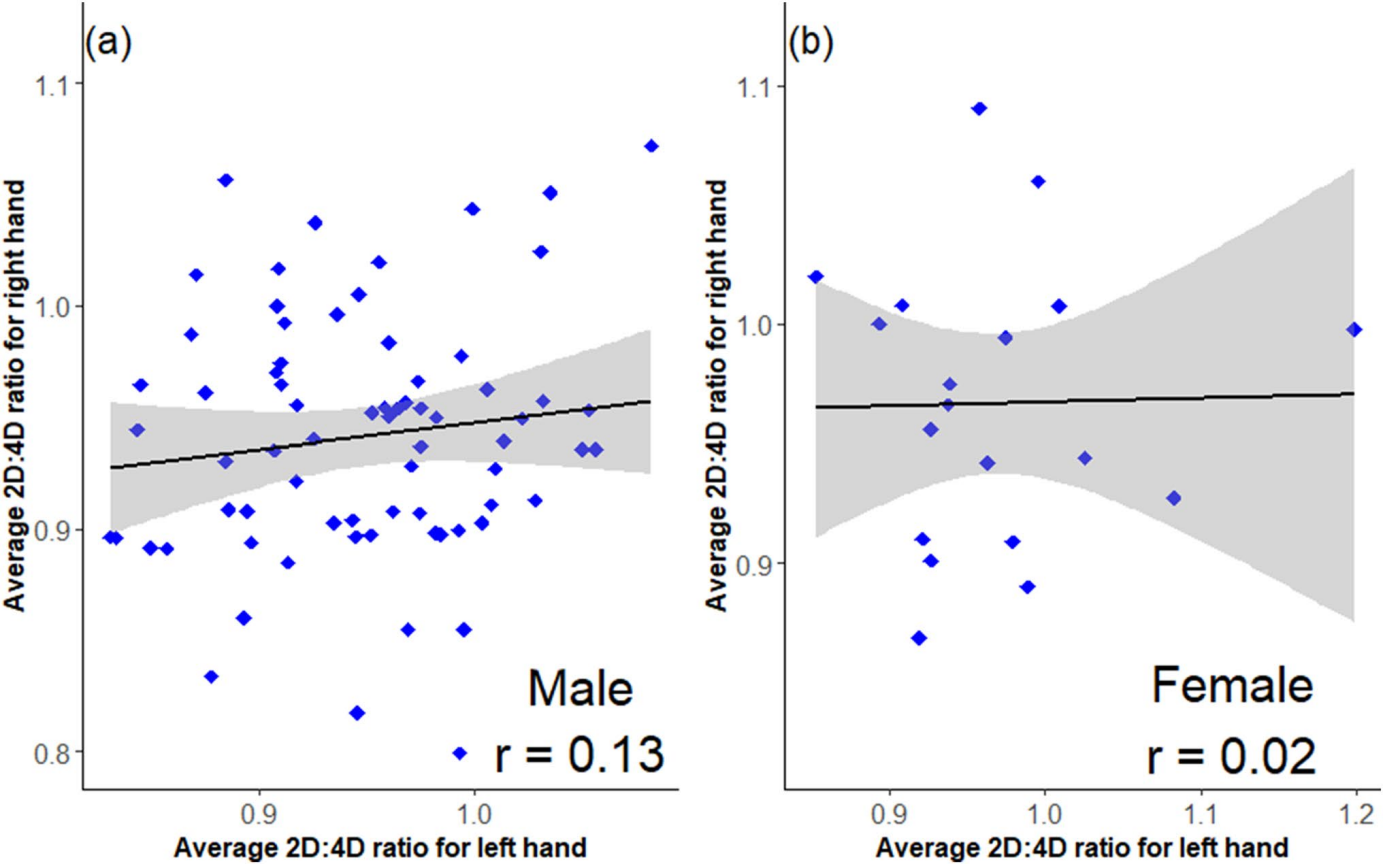
Scatterplot of average 2D:4D ratios of the left hand (X-axis) and right hand (Y-axis), for (**a**) Overall correlation between the average left-hand and right-hand 2D:4D in males (Pearson’s correlation; r = 0.13; *p* = 0.290) and (**b**) Overall correlation between average left hand and right hand 2D:4D in females (r = 0.02; *p* = 0.932) in the study population

**Fig. 2:**
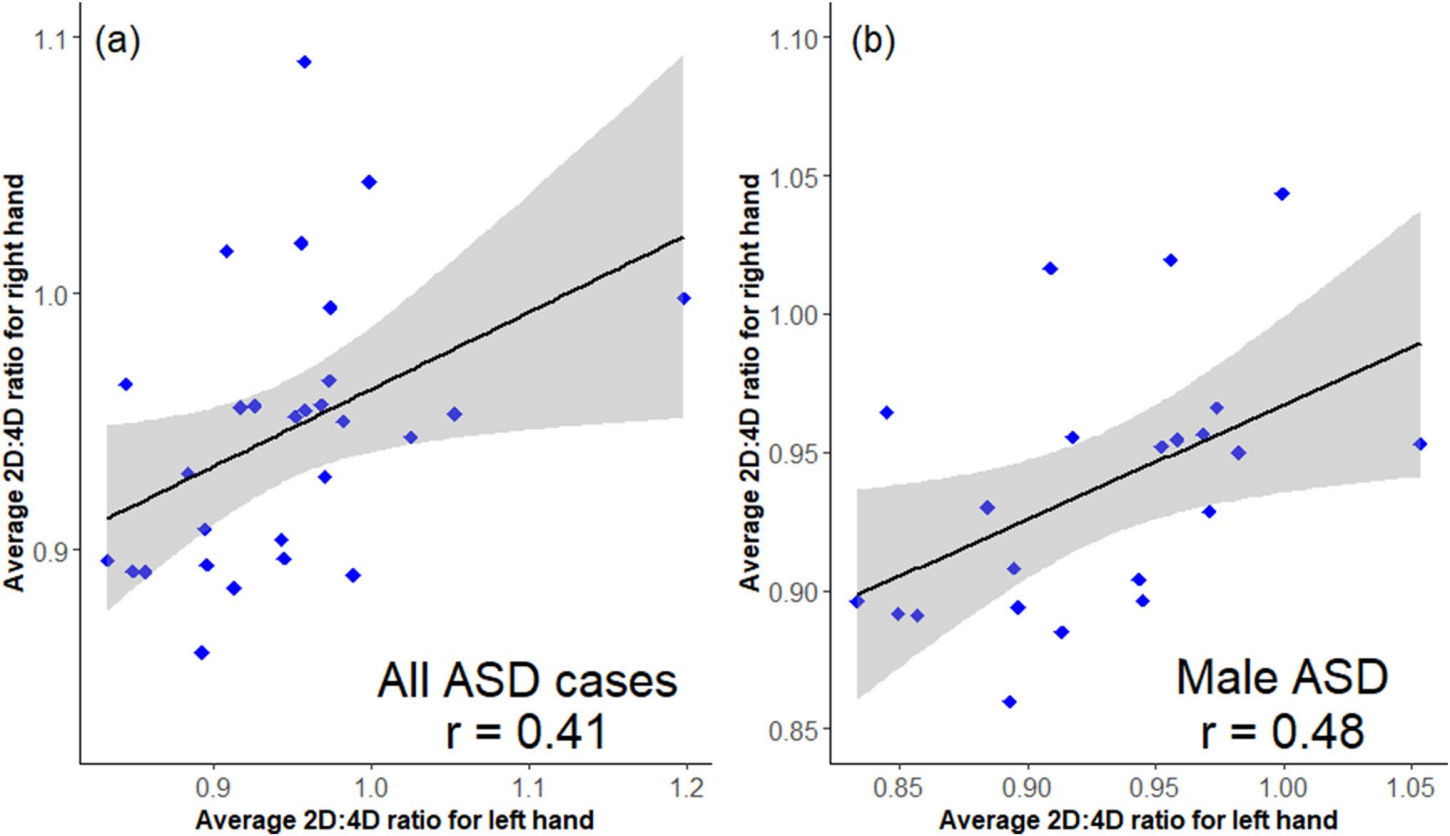
Scatterplot of average 2D:4D ratios of the left hand (X-axis) and right hand (Y-axis), for (**a**) Overall correlation between the average left-hand and right-hand 2D:4D for ASD cases (Pearson’s correlation; r = 0.41; *p* = 0.03) and (**b**) correlation between average left hand and right hand 2D:4D for male ASD cases only (r = 0.48; *p* = 0.023) in the study population

There is no correlation (and therefore, high asymmetry) between the average 2D:4D ratio of left hands and right hands in all study participants for males (Pearson’s correlation; r = 0.13, *p* = 0.290) and females (r = 0.02; *p* = 0.932) (Fig. 1a, b). However, there is a significant positive correlation between the average 2D:4D ratio of left and right hands among all ASD cases (males and females) (r = 0.41; 95% CI: 0.04–0.68, *p* = 0.030) (Fig. 2a) and in ASD males only (r = 0.48; 95% CI: 0.076–0.75, *p* = 0.023) (Fig. 2b). There is no correlation between the average 2D:4D ratio of left hands and right hands in TD males (r = − 0.002; *p* = 0.990), ASD females (r = 0.015; *p* = 0.978) and TD females (r = − 0.09; *p* = 0.780) (figures not shown).

Although there is no significant association between 2D:4D ratio and ASD among females, the adjusted ORs for females [Adjusted OR = 3.09 (95% CI: 0.98–19.86); left hand, and Adjusted OR = 1.23 (95% CI: 0.42–3.88); right hand] are generally higher than that in males (Table 2), suggesting possible positive relationship between 2D:4D ratio and ASD among females. Logistic regression (Table 2) revealed that for every 1 SD increase in the 2D:4D ratio for the left hand, there are lower odds of ASD diagnosis among males, after adjusting for ethnicity and age [Adjusted OR = 0.57 (95% Confidence Interval (CI): 0.31–0.96); *p* = 0.044]. In other words, male children with ASD tend to have low 2D:4D ratio for the left hand only, when compared to TD children. Such observation was not observed for the right hand of male children.

**Table 2:** Logistic regression of ASD diagnosis versus TD children, stratified by gender

Variables	Males (n = 71)				Females (n = 19)			
	Unadjusted OR (95% CI)	p-value	^Adjusted OR (95% CI)	p-value	Unadjusted OR (95% CI)	p-value	^Adjusted OR (95% CI)	p-value
2D:4D ratio (Left hand)	0.58 (0.32–0.97)	0.047*	0.57 (0.31–0.96)	0.044*	2.64 (0.90–13.89)	0.139	3.09 (0.98–19.86)	0.114
2D:4D ratio (Right hand)	0.90 (0.53–1.49)	0.674	0.87 (0.51–1.46)	0.602	1.38 (0.50–4.13)	0.535	1.23 (0.42–3.88)	0.702

The Odds Ratios (ORs) are for one unit change for 1 Standard Deviation (SD)

^Ethnicity and age at 2D:4D measurement were included in the model

*p-value is < 0.05

## Discussion

This study investigated the association between the 2D:4D ratio and ASD in Brunei Darussalam, and enrolled 62 TD and 28 ASD subjects, ranging in age from 3 to 11 years. Demographic information including age, gender and ethnicity of study participants were collected. Digital calliper measurements based on printed images of the palm (for 2D:4D length) were obtained to examine the association between the 2D:4D ratio and ASD in respective gender for left and right hands, separately.

Our data shows that about three quarters of participants were male (78.9%, n = 71) and of Malay ethnicity (75.6%, n = 68). For comparison, the demographic profile of Brunei is 66% Malays, with other ethnicities constituting 34% of the population; the male population is 51.4% (Ministry of Health B 2017). This suggests a predisposition for males to be diagnosed with ASD.

Our results show that for males, the likelihood of ASD diagnosis decreases as the 2D:4D ratio increases. This result may indirectly support the EMB theory of ASD, as high level of testosterone in males has been associated with lower 2D:4D ratio (Hönekopp et al. 2010). Previous studies which include male-only participants have shown an association between a lowered 2D:4D ratio association with ASD in right hands only (Krajmer et al. 2011; Noipayak 2009). However, it is important to note that in our study, this association is only observed in the left hand. A meta-analysis, comprising predominantly Caucasian population has shown that right-hand 2D:4D might be a more appropriate indicator of prenatal androgenisation than left-hand 2D:4D (Hönekopp et al. 2010). Population differences may influence the effect exerted by hormones on digit ratio to explain our study findings. In addition, a high SD for the 2D:4D ratio in our study population (SD range = 0.054 to 0.074) compared to that previously reported (SD range = 0.032 to 0.056) (Baharara et al. 2014) suggests higher variation in our population’s 2D:4D ratio compared to other population. This high variation may also be attributed to the small sample size in our study.

Unlike previous studies (Manning et al. 1998; McFadden and Shubel 2002), we did not observe any difference (*p* = 0.549) in the median 2D:4D ratios between the left hand of TD male children (0.962) and TD female children (0.938). For the right hand, although the median 2D:4D ratio for male children is comparatively lower than those of female children (0.940 versus 0.996, respectively), the difference is not significant (*p* = 0.303).

Although there was no significant association between 2D:4D ratio and ASD in females, the adjusted ORs for females are generally higher compared to males. This suggests that there may be a positive relationship between 2D:4D ratio and ASD diagnosis in females, contrary to findings in males. This may also imply different aetiologies for ASD in females (Lai et al. 2015). A study conducted in Japan on both genders reported an association between lowered 2D:4D ratio and ASD in right hands of adult males only (mean age ± SD = 29.7 ± 7.1 years), with the pattern reversed in females with ASD. The authors hypothesize that the higher 2D:4D ratio for adult females (mean age 25.9 ± 6.6 years) with ASDs might be due to alterations in fat tissue deposition around the digits to affect digit ratio (Masuya et al. 2015). Given our limited sample size, future studies with larger female sample size are necessary to validate this finding. Potential under-identification of females with ASD may occur due to biases in assessment tools/diagnostic practices, as well as exclusion of sociocultural factors to explain differential rates of diagnosis between genders (Kreiser and White 2014). Therefore, improved understanding of ASD in females to enhance gender-specific diagnosis.

It has been reported that handedness influences the ratio of 2D:4D ratio on the left and right hands (Gillam et al. 2008), where right-handers have higher right hand 2D:4D ratio than the left, with the pattern of asymmetry reversed in females—lower right hand 2D:4D ratio as compared to the left (Stoyanov et al. 2009). A meta-review from 12 studies has shown that the proportion of non-right handers (left-handers and mixed-handers) is elevated (60%) in ASD individuals (n = 497) (Rysstad and Pedersen 2016), compared to 10% non-right-handers in the general population (Coren and Porac 1977). In our study, participants’ handedness was not recorded therefore their effect and influence on our study findings remains to be ascertained.

Although our data show high asymmetry/ no signification correlation between the average 2D:4D ratio of left hands and right hands in all study participants (r = 0.13 and r = 0.02, for males and females respectively), there is a significant positive correlation between the average 2D:4D ratio of left hands and right hands of ASD males (r = 0.48; *p* = 0.023), suggesting a reduced asymmetry with a gender-specific effect (this correlation was not observed in ASD females). A matched case–control study has shown a reduced hemispheric asymmetry in the white matter of ASD compared to TD children and adolescents (Carper et al. 2016). The relationship between reduced brain asymmetry and diminished 2D:4D ratio asymmetry in ASD individuals remains to be determined. The development of hand preference requires significant exposure to a range of motor tasks, involving tools and other objects (Scharoun and Bryden 2014). As sensory and motor skills are common in individuals with ASD (Whyatt and Craig 2013; Thompson et al. 2017), the reduced asymmetry in left-hand versus right-hand 2D:4D ratio in ASD cases in our study may be attributed to a lack of motor and sensory skills and hence a delay in formation of a dominant/preferred hand or a lack of performance in both hands in ASD individuals, which may influence the growth and development of the digits to explain the reduced asymmetry of 2D:4D ratio between both hands. Food selectivity in ASD individuals (Bandini et al. 2010) as well as gastrointestinal disturbances (Jolanta Wasilewska and Klukowski 2015; Sanctuary et al. 2018; Sande et al. 2014) may also impact nutritional intake and absorption to affect overall growth and development, including digit growth and hence 2D:4D ratio.

Some of the study limitations include a small sample size (particularly for females) and a lack of record pertaining to the handedness of participants. Another limitation is the lack of record on the ASD subtype/severity of participants. It would be valuable to assess the relationship between 2D:4D ratio and ASD subtype/severity as subsequent evaluation of the heterogeneity of ASD severity among our study participants (if and given an association between 2D:4D ratio and ASD severity/subtypes) may elucidate some of the study findings. The non-response rate may also be improved as the non-responders may be of a different ASD subgroup(s) which may therefore affect our study findings. In addition, it was challenging to obtain palm photos from ASD individuals due to difficulties with them following instructions, as well as their limited attention span. These challenges have contrived us to utilizing digital photographic measurements over direct measurements. A study comparing measurement data from 2-dimensional digital photogrammetry method and direct anthropometry has concluded that both techniques are similarly valid and may replace each other (Franke-Gromberg et al. 2010), corroborating the accuracy and reliability of digital photographic measurement method (Franke-Gromberg et al. 2010; Cobb et al. 2011). Nevertheless, we strived to ensure consistency in 2D:4D measurements. Stringent quality control checks were performed to ensure clarity and consistency of angle/plane of photos before 2D:4D measurements were taken which is reflected in the high ICC values (left hand = 0.98; right hand = 0.97), indicating high 2D:4D measurement similarity between raters. The measurements were also performed blindly by two independent raters with gender, age and ethnicity of the participants anonymised, to prevent introduction of bias to the result.

## Conclusions

This study shows an association between a lowered 2D:4D ratio of the left hand and ASD diagnosis in ASD males. No significant association between 2D:4D ratio and ASD was observed in female participants for the left or right hand, which may be due to differing aetiology for ASD in females. These findings require further validation, due to the small sample size. Upon validation with a larger sample size, the 2D:4D ratio may be a physical biomarker for diagnosis to complement diagnosis. A shortened time to diagnosis is essential as it allows early implementation of appropriate intervention and management. Finally, this study may serve as a platform for prospective ASD-related research studies of common interest to the community and researchers, to propel understanding of the aetiology of ASD, improve diagnosis for early intervention and management.

## Supplementary Information

Below is the link to the electronic supplementary material.

Supplementary file1 (DOCX 1137 KB)

## Data Availability

The dataset used and/or analysed during the current study are available from the corresponding author upon reasonable request.

## Abbreviations

2D:4D ratio: Second digit/index finger to Fourth digit/ring finger ratio
ASD: Autism spectrum disorders
EMB: Extreme male brain
FT: Foetal testosterone
PCOS: Polycystic ovarian syndrome
TD: Typically developing
