# Supplementary material for: 2D:4D Ratio and Autism Spectrum Disorder in Brunei Darussalam
Source: J Autism Dev Disord. 2021 Feb 11;51(12):4577–86. doi: 10.1007/s10803-021-04899-9 (PMC8531075; doi:10.1007/s10803-021-04899-9)
Supplement: Supplementary file 1 — Supplementary file1 (DOCX 1137 KB) [file 10803_2021_4899_MOESM1_ESM.docx]

**Supplementary Figure 1 (S1):**


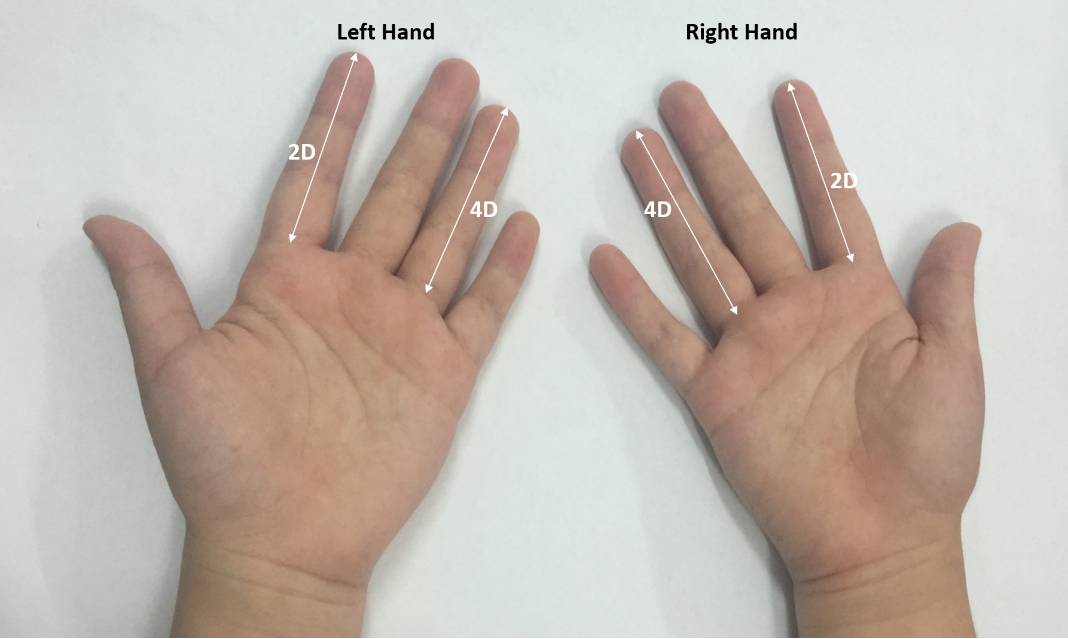


S1. A sample of palm photo obtained from one of the study participants for 2D:4D measurement and analysis
